# Medicare Part D Coverage of Drugs Selected for the Drug Price Negotiation Program

**DOI:** 10.1001/jamahealthforum.2023.5237

**Published:** 2024-02-09

**Authors:** Julie A. Patterson, Tyler D. Wagner, John M. O’Brien, Jonathan D. Campbell

**Affiliations:** 1 National Pharmaceutical Council, Washington, DC

## Abstract

This cross-sectional study describes and historically benchmarks Medicare Part D coverage in 2019 and 2023 for the first 10 drugs selected for negotiation.

## Introduction

The Inflation Reduction Act (IRA) created a Drug Price Negotiation Program for selected drugs and requires Medicare formulary coverage of selected drugs once a maximum fair price is in effect. Because Medicare is not requiring specific tiering or utilization management for selected drugs in 2026,^1^ the effect of selection on formulary tiering and restrictions is not known.^2,3^ However, given the role of drug rebates in plan coverage decisions, plan sponsors may be incentivized to place selected drugs on less favorable tiers or apply utilization management to steer beneficiaries away from selected drugs^1^ if maximum fair prices reduce rebates while nonselected drugs in the same class retain the flexibility for higher rebates. This cross-sectional study aimed to describe and historically benchmark Medicare Part D coverage in 2019 and 2023 for the first 10 drugs selected for negotiation.

## Methods

We analyzed Medicare formulary and pricing files for Part D plans for the first quarters of 2019 and 2023, excluding special needs plans and plans with suppressed data and/or fewer than 10 enrollees. We described formulary coverage of the 10 drugs, and, when covered, utilization management and tiering. Specifically, we calculated the proportion of plans that covered at least 1 dosage form of a selected drug, identified by RxNorm concept unique identifiers, without prior authorization or step therapy, and placed at least 1 dosage form on tiers 1 to 3 (ie, preferred brand name drug or better).^4^ In a supporting analysis, estimates were weighted by plan enrollment for beneficiary-level access to selected drugs. All data were publicly available and not considered human participant research. This study followed STROBE reporting guidelines.

## Results

Among the 10 drugs, average coverage was generally high in 2019 (3345 plans [82.0%]) and further increased in 2023 (4362 plans [91.3%]), with coverage numerically improving over the study period for 3 of the 4 drugs with the lowest coverage in 2019: ustekinumab (31.6% to 70.1%), dapagliflozin (60.8% to 88.6%), and etanercept (71.8% to 99.2%) (Table 1). Access without prior authorization was common for cardiovascular and diabetes medications (2019: 71.2%-100%; 2023: 99.8%-100%) but rare for biologic drugs etanercept and ustekinumab as well as oncology drug ibrutinib in both 2019 (1.5%-2.7%) and 2023 (0.2%-1.7%). In contrast, access without step therapy was high across all 10 selected drugs in both study years (2019: 92.1%-100%; 2023: 94.6%-100%).

**Table 1.** Medicare Plan Coverage of Part D Medications Selected for the Centers for Medicare & Medicaid Services’ Drug Price Negotiation Program for Initial Price Applicability Year 2026^a^

Medication	% Covering		% With access to ≥1 dose without prior authorization^b^		% With access to ≥1 dose without step therapy^b^		Lowest tier (1, 2, or 3), %^b^	
	2019	2023	2019	2023	2019	2023	2019	2023
Apixaban	98.9	100	100	100	100	99.2	94.2	95.3
Empagliflozin	97.7	99.8	99.5	100	94.4	99.8	99.8	97.9
Rivaroxaban	98.7	99.5	100	100	100	100	98.0	98.1
Sitagliptin	96.5	94.9	99.6	100	96.8	98.7	97.9	97.8
Dapagliflozin	60.8	88.6	99.2	99.8	97.4	99.9	65.6	81.5
Sacubitril/valsartan	100	100	71.2	100	100	100	91.5	99.7
Etanercept	71.8	99.2	2.7	1.6	99.9	100	2.9	0.7
Ustekinumab	31.6	70.1	1.5	0.2	99.7	100	1.2	0.5
Ibrutinib	100	100	2.4	1.7	100	100	1.6	0.7
Insulin aspart	63.5	61.0	100	100	92.1	94.6	89.3	94.0

^a^ Data included were analyzed for the first quarter of 2019 and 2023 from the Prescription Drug Plan Formulary, Pharmacy Network, and Pricing Information Files of the Centers for Medicare & Medicaid Services.

^b^ Specific aspects of Medicare plan coverage (utilization management and tiering) are reported among plans that offered coverage of the selected drug.

Among cardiovascular and diabetes medications, placement of at least 1 dosage form on tiers 1 to 3 was common (2019: 65.6%-99.8%; 2023: 81.5%-99.7%). Lower-tier placement was rare in 2019 for etanercept, ustekinumab, and ibrutinib (1.2%-2.9%) and less than 1% of plans for all 3 drugs in 2023. Tier placement numerically increased for dapagliflozin (65.6% to 81.5%), sacubitril/valsartan (91.5% to 99.7%), and insulin aspart (89.3% to 94.0%) over the study period. Results from the beneficiary-level supporting analysis were generally consistent with the plan-level findings (Table 2).

**Table 2.** Weighted Percentages of Beneficiaries With Medicare Plan Coverage of Part D Medications Selected for the Centers for Medicare & Medicaid Services’ Drug Price Negotiation Program for Initial Price Applicability Year 2026^a^

Medication	% Covering		% With access to ≥1 dose without prior authorization^b^		% With access to ≥1 dose without step therapy^b^		Lowest tier (1, 2, or 3), %^b^	
	2019	2023	2019	2023	2019	2023	2019	2023
Apixaban	97.9	100	100	100	100	97.9	94.1	87.5
Empagliflozin	98.4	99.9	99.9	100	97.9	100	100	91.7
Rivaroxaban	99.3	99.8	100	100	100	100	97.1	96.6
Sitagliptin	93.1	93.4	99.8	100	98.7	99.4	96.8	91.1
Dapagliflozin	67.9	89.7	99.8	99.8	97.8	99.9	62.6	70.7
Sacubitril/valsartan	100	100	72.1	100	100	100	95.5	99.7
Etanercept	62.3	97.9	5.0	3.1	100	100	1.5	0.8
Ustekinumab	23.5	65.6	2.2	0.2	99.4	100	1.6	0.6
Ibrutinib	100	100	3.1	3.1	100	100	1.3	0.8
Insulin aspart	64.3	59.9	100	100	97.4	98.3	92.8	98.0

^a^ Data included were analyzed for the first quarter of 2019 and 2023 from the Prescription Drug Plan Formulary, Pharmacy Network, and Pricing Information Files of the Centers for Medicare & Medicaid Services and Centers for Medicare & Medicaid Services enrollment data for 2019 and 2023.

^b^ Specific aspects of Medicare plan coverage (utilization management and tiering) are reported among plans that offered coverage of the selected drug.

## Discussion

In this cross-sectional study, among drugs selected for Medicare price negotiation, beneficiary access—including formulary coverage, access without utilization management, and favorable tier placement—was often high in 2019 and 2023. These findings support concerns about future access waning through increased utilization management and adverse tiering, which, consistent with past research,^5,6^ were already common for selected biologic and oncology drugs. While the IRA’s formulary inclusion requirement will improve access to selected drugs previously excluded from plan formularies (eg, ustekinumab and insulin aspart), considerable uncertainty remains about how the Centers for Medicare & Medicaid Services will “monitor practices that may undermine enrollee access to selected drugs”^1^ and how actionable the formulary review process alone can be in protecting meaningful access.

As a limitation, this study focused on tiering and utilization management, rather than cost-sharing structures. The IRA’s Part D out-of-pocket cap ($2000 in 2025) may improve patient access, particularly for both selected and nonselected drugs placed on specialty tiers. The perverse incentives for utilization management and adverse tiering of drugs selected for the Drug Price Negotiation Program warrant additional monitoring of patient access as well as consideration of preventive measures to ensure that patient access to selected drugs is not reduced.

## Notes

Supplement. Data Sharing Statement
