# Supplementary material for: Medicare Part D Coverage of Drugs Selected for the Drug Price Negotiation Program
Source: JAMA Health Forum. 2024 Feb 9;5(2):e235237. doi: 10.1001/jamahealthforum.2023.5237 (PMC10858397; doi:10.1001/jamahealthforum.2023.5237)
Supplement: Supplement. — Data Sharing Statement [file jamahealthforum-e235237-s001.pdf]

Data Sharing Statement

Patterson. Medicare Part D Coverage of Drugs Selected for the Drug Price Negotiation Program. *JAMA Health Forum*. Published February 09, 2024. doi:10.1001/jamahealthforum.2023.5237

Data
Data available: No

Additional Information
Explanation for why data not available: Publicly available data; https://www.cms.gov/data-research/statistics-trends-and-reports/prescription-drug-plan-formulary-pharmacy-network-and-pricing-information-files-download
